# Machine learning approach to predict body weight in adults

**DOI:** 10.3389/fpubh.2023.1090146

**Published:** 2023-06-15

**Authors:** Kazuya Fujihara, Mayuko Yamada Harada, Chika Horikawa, Midori Iwanaga, Hirofumi Tanaka, Hitoshi Nomura, Yasuharu Sui, Kyouhei Tanabe, Takaho Yamada, Satoru Kodama, Kiminori Kato, Hirohito Sone

**Affiliations:** ^1^Department of Endocrinology and Metabolism, Faculty of Medicine, Niigata University, Niigata, Japan; ^2^Department of Health and Nutrition, Faculty of Human Life Studies, University of Niigata Prefecture, Niigata, Japan; ^3^NEC Solution Innovators, Ltd., Tokyo, Japan; ^4^Department of Prevention of Noncommunicable Diseases and Promotion of Health Checkup, Niigata University, Niigata, Japan

**Keywords:** body weight, prediction, machine learning model, heterogeneous mixture learning technology, body mass index

## Abstract

**Background:**

Obesity is an established risk factor for non-communicable diseases such as type 2 diabetes mellitus, hypertension and cardiovascular disease. Thus, weight control is a key factor in the prevention of non-communicable diseases. A simple and quick method to predict weight change over a few years could be helpful for weight management in clinical settings.

**Methods:**

We examined the ability of a machine learning model that we constructed to predict changes in future body weight over 3 years using big data. Input in the machine learning model were three-year data on 50,000 Japanese persons (32,977 men) aged 19–91 years who underwent annual health examinations. The predictive formulas that used heterogeneous mixture learning technology (HMLT) to predict body weight in the subsequent 3 years were validated for 5,000 persons. The root mean square error (RMSE) was used to evaluate accuracy compared with multiple regression.

**Results:**

The machine learning model utilizing HMLT automatically generated five predictive formulas. The influence of lifestyle on body weight was found to be large in people with a high body mass index (BMI) at baseline (BMI ≥29.93 kg/m^2^) and in young people (<24 years) with a low BMI (BMI <23.44 kg/m^2^). The RMSE was 1.914 in the validation set which reflects ability comparable to that of the multiple regression model of 1.890 (*p* = 0.323).

**Conclusion:**

The HMLT-based machine learning model could successfully predict weight change over 3 years. Our model could automatically identify groups whose lifestyle profoundly impacted weight loss and factors the influenced body weight change in individuals. Although this model must be validated in other populations, including other ethnic groups, before being widely implemented in global clinical settings, results suggested that this machine learning model could contribute to individualized weight management.

## Introduction

Obesity is an established risk factor for non-communicable diseases such as type 2 diabetes mellitus, hypertension and cardiovascular disease (1–4). Lifestyle interventions involving weight loss have reduced the incidence of non-communicable diseases (5–7). A combined approach using dietary habits and physical activity is important for both short- and long-term.

Except for a few studies (8, 9), it has been shown that weight loss is difficult to achieve. The Trials of Hypertension Prevention (TOHP-II) showed that only 43% of participants lost more than 4 kg during a 6-month intensive behavioral intervention and that the drop-out rate was relatively high (10). Similarly, only 25% of participants lost at least 5% of their bodyweight between baseline and 12 months in an intervention conducted in primary care settings (11). These findings revealed the limitations of uniform treatment for weight control and that factors that influence weight vary substantially among individuals. Thus, an individualized approach to lifestyle interventions is essential for weight management.

Even when metabolic health was maintained over long periods, obesity was shown to remain a risk factor for cardiovascular disease (12). Thus, a simple and quick method to predict weight change over a few years could be helpful in weight management. In addition, not only identifying groups whose lifestyle profoundly impacts weight control but also clarifying factors that strongly influence an individual’s body weight is useful in clinical settings.

Machine learning, which can determine patterns and decision rules from data (13–16), is rapidly becoming used in clinical practice. The applications of machine learning for the early detection of diabetes, including its complications, and cancer that provided clear-cut diagnostic gold standards have been evaluated (17–24). However, little is known about the usefulness of machine learning to predict body weight over the long term, as well as to identify factors that influence body weight in individuals. Moreover, some issues involving the nature of the machine learning algorithm, which is often referred to as a black box model (13–16), could be a barrier to the practice of evidence-based medicine.

Heterogeneous mixture learning technology (HMLT) is a newly-developed method to automatically divide original data to increase the mining accuracy of patterns, trends, and rules in the data (25). Even when it is difficult to know the number of splits or what clues to use for splitting, it is possible to conduct appropriate splits at high speed based on characteristics of the new groups (25). Thus, this technology be expected to be superior to other machine learning models in predicting weight change. Although unexplained machine learning based weight prediction models for relatively short periods of time have been reported (26, 27), little is known about the effectiveness of explainable machine learning in predicting weight change over a relatively long period of time.

Therefore, we constructed a machine learning model to predict body weight over 3 years using medical checkup data. Using this information, we attempted to evaluate the ability of machine learning models for weight prediction and to identify factors that influence weight loss in individuals.

## Methods

### Study participants

Study participants were 67,021 Japanese individuals who underwent an initial annual health examination between April 2014 and January 2020 at the Niigata Association of Occupational Health in Niigata, Japan. The Niigata Association of Occupational Health has multiple health management centers in Niigata Prefecture and has been conducting screening health examinations and routine health examinations. The ethics committee of Niigata University approved the present study (2020–0003). Informed consent was obtained from all patients at each participating institute in accordance with the Guidelines for Epidemiological Studies of the Ministry of Health, Labour and Welfare of Japan (28, 29). Individuals with missing data for in the variables analyses were excluded (*n* = 12,021). Finally, this study included 55,000 Japanese with health examination data.

### Assessment of clinical variables

Body mass index was calculated from height and weight measured without shoes or heavy clothing. Smoking and alcohol intake status, exercise habit, physical activity, skipping breakfast, walking speed, eating speed, and history of dyslipidemia, diabetes, and hypertension were assessed using a self-report questionnaire at each annual examination. Blood samples were collected after an overnight fast. The National Glycohemoglobin Standardization Program equivalent value (%) was used to convert the HbA1c value.

### Machine learning model utilizing HMLT

We used HMLT to predict future body weight. It is a type of machine learning algorithm developed by Nippon Electric Company (25). It applies factorized asymptotic Bayesian theory to data with heteroscedasticity (30–32), which is a mixture of data that follows different patterns and rules, to find factors that set the rules and eliminate inherent heteroscedasticity (Supplementary Figure S1). The HMLT has the following features: (i) high predictive accuracy and high interpretability by the model and (ii) previous used in energy demand forecasting systems and demand forecasting automatic ordering systems. For example, for the hypothesis that the data contain a mixture of nonlinear and linear relationships (Supplementary Figure S1A), HMLT can divide the data into two groups (Supplementary Figure S1B). However, for the hypothesis of a mixture of multiple linear relationships (Supplementary Figure S1C), HMLT automatically generates three groups. We let HMLT learn the trends within the health checkup data over 3 years and generate equations to predict body weight in the last year using data from the first 2 years. Explanatory variables were weight, baseline characteristics, and lifestyle factors. To predict weight after 3 years, the weight after 1 year was first predicted. Then, based on the weight at 1 year the weight after 2 years was predicted. Similarly, the weight after 3 years was predicted using information on body weight for 2 years. For lifestyle issues, the user selected the input data for each lifestyle category after 1 year, which would continue without change for 3 years. Input in the machine learning model were 3-year data on 50,000 persons and the model was validated by 5,000 persons.

### Statistical analysis

Categorical variables were expressed as numerals and percentages and were compared with χ^2^ tests. Continuous variables were expressed as mean ± SD and the Student’ *t-*test was used for comparisons in each group. The root mean square error (RMSE) was used to evaluate accuracy compared to a multiple regression model. RMSE is a value obtained by averaging the square of the error and taking its square root. The formula for calculating RMSE is $\mathrm{RSME}=\sqrt{\frac{1}{n}\overset{n}{\underset{k=1}{\sum }}{\left(y_{i}-{\bar{y}}_{i}\right)}^{2}}$ (33, 34). All statistical analyses were performed by scipy.stats (version 1.5.4, chi2_contingency, ttest_ind). Statistical significance was considered for *p* < 0.05.

## Results

Characteristics of participants according to learning/validation data and sex are shown in Table 1. Mean age was 48 years and 67% were men. There were no significant differences between learning data and validation data except for height in men.

**Table 1:** Characteristics of study participants.

	Learning data		Validation data	
	Male	Female	Male	Female
*n*	32,977 (66)	17,023 (34)	3,368 (67)	1,632 (33)
Age (year)	48 ± 12	48 ± 12	48 ± 12	48 ± 12
Height(cm)	170.8 ± 6.2	157.8 ± 5.7	171.0 ± 6.2	158.0 ± 5.8
Weight (kg)	68.3 ± 11.1	54.4 ± 9.6	68.5 ± 11.3	54.1 ± 9.7
Body mass index (kg/m^2^)	23.4 ± 3.4	21.8 ± 3.7	23.4 ± 3.5	21.7 ± 3.7
Waist circumstance (cm)	83.5 ± 9.3	78.4 ± 9.6	83.6 ± 9.4	78.6 ± 10.4
Systolic blood pressure (mmHg)	124 ± 16	117 ± 16	125 ± 16	117 ± 16
Diastolic blood pressure (mmHg)	78 ± 11	72 ± 11	78 ± 11	72 ± 11
HbA1c (%)	5.6 ± 0.5	5.6 ± 0.4	5.6 ± 0.5	5.6 ± 0.4
TG (mg/dL)	122 ± 79	87 ± 51	122 ± 78	86 ± 50
HDL cholesterol (mg/dL)	59 ± 15	70 ± 16	59 ± 15	70 ± 15
LDL cholesterol (mg/dL)	123 ± 30	119 ± 30	123 ± 30	119 ± 30
Fasting blood glucose (mg/dL)	99 ± 14	94 ± 11	100 ± 15	93 ± 12
AST (U/L)	25 ± 10	21 ± 7	25 ± 10	21 ± 7
ALT (U/L)	26 ± 16	16 ± 9	25 ± 15	16 ± 9
γ-GTP (U/L)	49 ± 43	25 ± 20	50 ± 45	26 ± 22

Data given as n (%), mean ± SD. AST, aspartate aminotransferase; ALT, alanine aminotransferase; HDL- cholesterol, high-density lipoprotein cholesterol; LDL-C, low-density lipoprotein cholesterol; TG, triglycerides.

**Figure 1:**
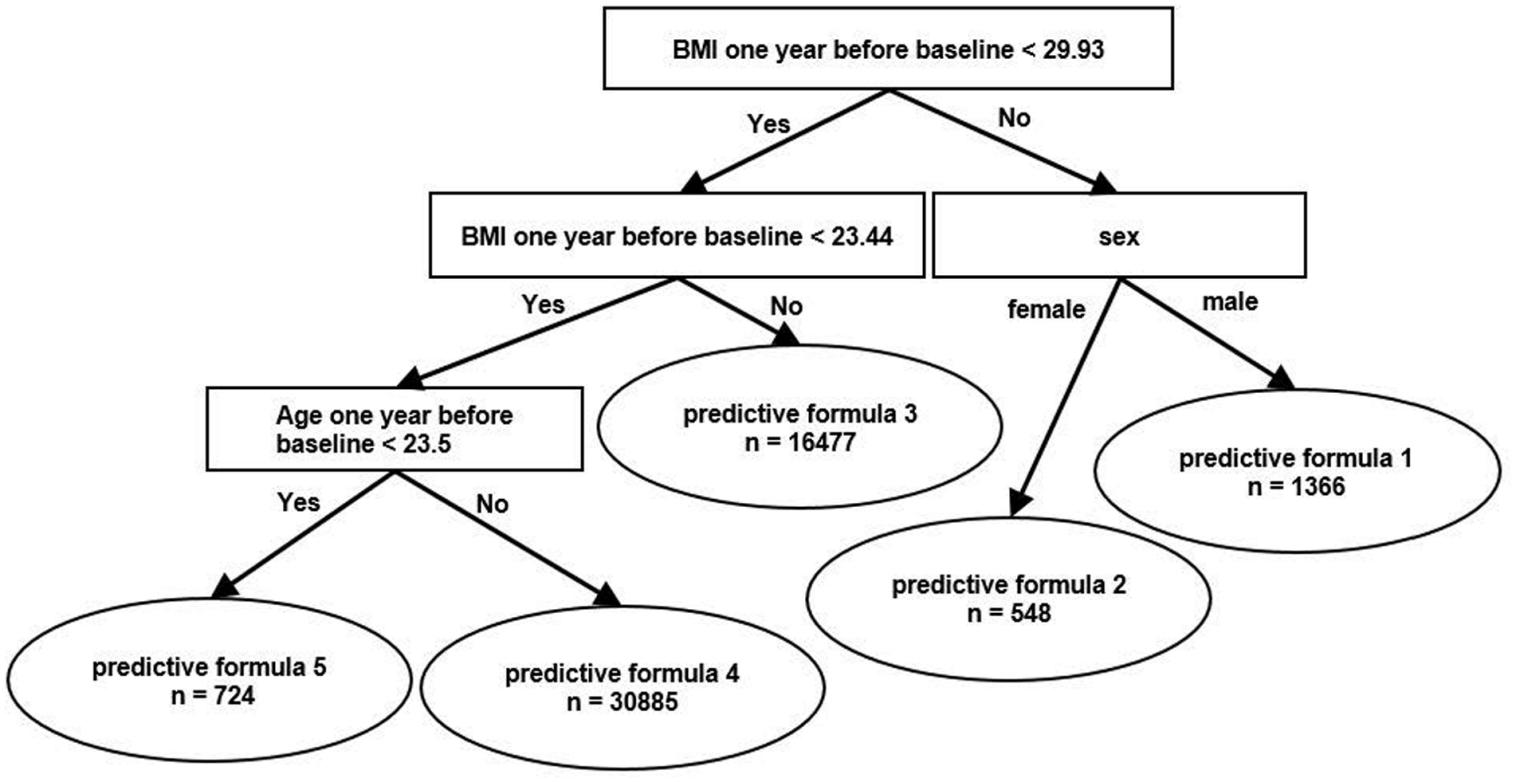
Schema of branches of the predictive formula using heterogeneous mixture learning models. BMI, body mass index.

Figure 1 is a schema of branches of the predictive formulas that were produced from a data of information on participants using the HMLT model. In our case, HMLT automatically generated 5 formulas by a linear model based on a rule that maximizes the predictive capacity throughout the dataset. As a result, participants were classified into one of the following five categories, i.e., predictive formulas, according to the algorithm shown in the Figure 1. First, the population was stratified into two groups according to BMI <29.93 kg/m^2^ and BMI ≥29.93 kg/m^2^. Then, each group was stratified by BMI ≥ 23.44 kg/m^2^ and BMI <23.44 kg/m^2^ or sex (men/women). The group with BMI ≥23.44 kg/m^2^ was further divided into two groups depending on whether they were ≤24 years of age or >24 years of age. Finally, people were divided into five groups.

Table 2 shows the predictive formula for each of the 5 categories according to the algorithm shown in Figure 1. Each of the coefficients is a coefficient of the linear model used to calculate weight at the predicted year by baseline weight and lifestyle changes. The coefficients were relatively high in formulas 1, 2, and 5. RMSE was 1.914, which reflects ability comparable to the multiple regression model and was 1.890 (*p* = 0.323; Supplementary Table S1).

**Table 2:** Coefficient of each variable in each predictive formula in five categories according to the algorithm.

Explanatory variables	Intention to improve lifestyle *			Predictive formula				
	1	2	3	1	2	3	4	5
Weight at baseline				1.00	1.00	1.00	1.00	1.00
Difference in weight between 1 year before baseline and baseline				−0.14	−0.13	−0.19	−0.21	−0.22
Height at baseline				0.01	0.03	0.00	0.01	0.02
Sex (male)				–	–	–	–	–
Age at baseline				−0.02	–	−0.02	−0.01	–
Interview at baseline. Do you want to use the opportunity of health instructions for improvement of your life habits? (No)				–	–	–	–	–
Interview at predictive year. Do you want to use the opportunity of health instructions for improvement of your life habits? (Yes)				−0.11	−0.06	–	−0.03	–
Interview at baseline. Are you a heavy smoker? (No)				−0.29	−0.45	–	–	−0.92
Interview at predictive year. Are you a heavy smoker? (No)	〇			−0.11	−0.30	–	–	−0.79
		〇		–	–	−0.11	–	−0.79
			〇	–	−1.15	−0.16	−0.02	–
Interview at baseline. Drinking frequency and quantity				−0.00	−0.01	−0.01	−0.00	−0.01
Interview at predictive year. Drinking frequency and quantity				0.01	0.03	0.00	0.00	0.01
Interview at baseline. Is your walking speed faster than that of others your age and sex? (No)				−0.02	−0.26	−0.09	−0.02	–
Interview at predictive year. Is your walking speed faster than that others your age and sex? (Yes)	〇			−0.13	−0.01	−0.03	−0.04	−0.03
		〇		–	−0.03	−0.03	−0.00	−0.37
			〇	−0.07	−0.59	–	–	−0.08
Interview at baseline. Do you walk or have any equivalent amount of physical activity more than 1 h a day in your daily life? (No)				−0.34	−0.20	−0.14	−0.06	−0.27
Interview at predictive year. Do you walk or have any equivalent amount of physical activity more than 1 h a day in your daily life? (Yes)	〇			−0.36	−0.35	−0.13	−0.11	−0.25
		〇		−0.01	−0.07	−0.03	−0.03	–
			〇	–	–	−0.10	−0.06	−1.79
Interview at baseline. Do you perform exercise of moderate intensity at least twice a week for 30 min over a period of 1 year? (No)				−0.36	–	−0.25	−0.11	−0.04
Interview at predictive year. Do you perform exercise of moderate intensity at least twice a week for 30 min over a period of 1 year? (Yes)	〇			−0.61	–	–	–	−0.15
		〇		−0.74	–	−0.05	–	−0.58
			〇	−0.42	–	–	−0.01	–
Interview at predictive year. Do you perform exercise of moderate intensity at least twice a week for 30 min over a period of 1 year? (No)	〇			0.09	–	0.24	0.11	–
			〇	–	–	–	–	–
Interview at baseline. Do you skip breakfast more than 3 times a week? (Yes)				−0.18	–	–	–	−0.04
Interview at predictive year. Do you skip breakfast more than 3 times a week? (No)	〇			−0.24	–	–	−0.09	−0.32
		〇		−0.24	−0.50	−0.06	−0.01	-
			〇	−0.67	–	−0.13	−0.01	−0.11
Interview at baseline. Is your eating speed quicker than others? (quicker)				–	−0.39	–	−0.01	−0.41
Interview at baseline. Is your eating speed quicker than others? (normal)				−0.08	0.07	0.04	0.03	−0.01
Interview at predictive year. Is your eating speed quicker than others? (normal)	〇			−0.34	−0.35	−0.04	−0.07	−0.52
		〇		0.06	0.27	−0.19	−0.15	−1.48
			〇	−0.67	0.31	−0.06	−0.03	−0.33
Interview at predictive year. Is your eating speed quicker than others? (no)	〇			−1.07	–	−0.01	−0.14	−0.75
		〇		–	−0.22	−0.00	−0.03	−1.65
			〇	−1.84	−1.12	–	−0.00	–
Interview at baseline. Do you eat supper 2 h before bedtime more than 3 times a week? (Yes)				−0.28	−0.63	−0.10	–	–
Interview at predictive year. Do you eat supper 2 h before bedtime more than 3 times a week? (No)	〇			−0.02	−0.39	−0.16	−0.07	−0.21
		〇		−0.61	–	–	−0.11	−0.30
			〇	−0.61	−0.03	−0.16	−0.19	–
Interview at baseline. Do you eat snacks or drink sweet beverages between meals? (Yes)				−0.15	−0.02	−0.21	−0.00	−0.49
Interview at predictive year. Do you eat snacks or drink sweet beverages between meals? (No)	〇			−0.35	−0.12	−0.16	−0.05	−0.29
		〇		−0.47	−1.72	−0.13	−0.00	−0.28
			〇	−0.26	−1.45	−0.18	−0.11	−1.30
Interview at baseline. Do you sleep well? (No)				−0.22	−0.31	–	–	−0.09
Interview at predictive year. Do you sleep well? (Yes)	〇			−0.11	−0.29	–	–	–
		〇		−0.37	–	−0.07	−0.07	−0.33
			〇	–	–	−0.12	−0.02	–
Bias				2.12	−2.34	1.57	0.16	0.18

*Do you want to improve your life habits such as eating and exercise? 1: No, 2: already trying to improve (less than 6 months), 3: already trying to improve (over 6 months).

**Figure 2:**
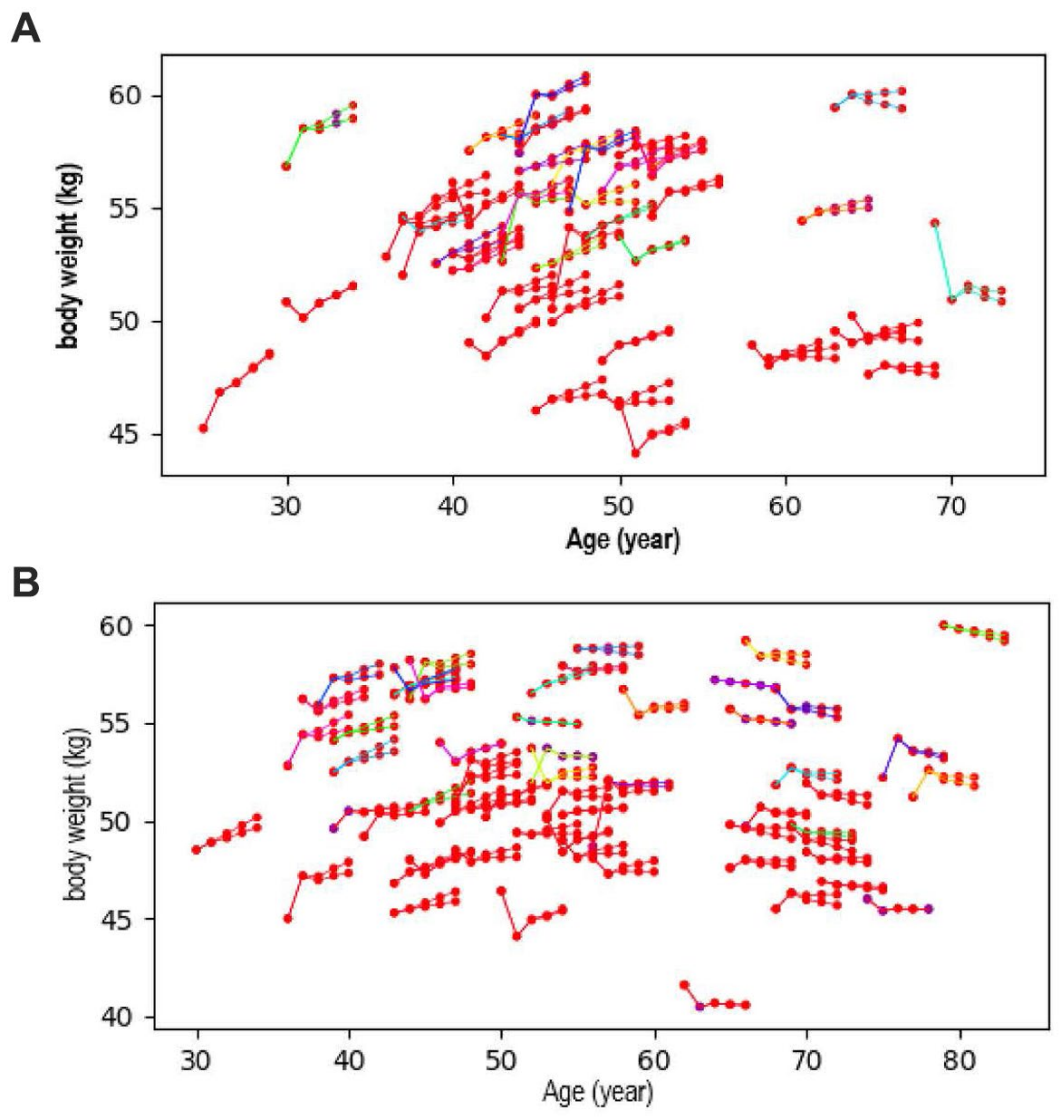
Trend of future body weight according to **(A)** breakfast intake (yes or no) and **(B)** walking speed (fast or not). The first two dots show measured values, and the latter three dots show predicted values for body weight.

Figure 2 shows the examples of trends of future body weight according to Figure 2A breakfast intake (yes or no) and Figure 2B walking speed (fast or not). The first two dots of each component show actual measured values for the first 2 years and the latter three dots show predicted values of body weight for the third year of the follow-up. People with healthy lifestyle habits, such as eating breakfast or fast walking, tended to experience weight reduction, while unfavorable lifestyle habits were shown to increase weight (Figure 2). Similar body weight trends were observed for other lifestyle factors.

## Discussion

We constructed a machine learning model to predict body weight over 3 years using medical checkup data. The machine learning model utilizing HMLT automatically generated five predictive formulas and the predictive ability of HMLT was similar to that of multiple regression. Also, HMLT automatically identified groups of people whose lifestyle habits had a profound impact on weight loss.

To our knowledge, this was the first trial to predict body weight over 3 years using artificial intelligence, which resolved the issue of the explainable predictions in a way that humans can understand. The machine learning model could predict weight change over a 3-year period. Those findings suggested that the machine learning model could contribute to an individualized approach to weight management in clinical settings.

Weight loss is a key factor in the prevention of non-communicable diseases. Body weight was shown to gradually increased before the onset of diabetes regardless of obesity level (35). The Diabetes Prevention Program showed that weight loss through lifestyle interventions was the dominant predictor of reduced diabetes incidence. In that study, there was a 16% reduction in risk for every kilogram of weight loss. It was also shown that weight reduction was associated with significant health benefits in Japanese (36, 37). However, weight loss is difficult to achieve, and about a half of participants failed to lose more than 4 kg during a 6-month intensive behavioral intervention with a relatively high drop-out rate (10). Although lifestyle factors were shown to significantly influence body weight (38, 39), the impact of each component of an individual’s lifestyle on weight loss was complicated and, moreover, varied widely among individuals. In our study, the coefficients were relatively high for formulas 1, 2, and 5 (Table 2). In other words, the influence of lifestyle on body weight was found to be large in people with a high BMI at baseline and in younger people with a low BMI in our cohort, which was not possible with conventional multiple regression analysis. An approach using machine learning may make it possible to identify those for whom particular lifestyle interventions in each regional (or ethnic) population would be appropriate, leading to a more effective use of limited medical resources.

A quick way to predict future weight change as well identify factors that influence weight change in individuals has been eagerly awaited in clinical practice. Although previous research provided several models to estimate individual weight change using dietary intake and/or physical activity (40, 41), few models have visualized details on which of these influence an individual’s weight change over the subsequent few years. Our findings made it possible to simulate an individual’s weight change accompanied by lifestyle changes at least over 3 years that was accompanied by lifestyle changes (Supplementary Figure S2). Thus, individualized instructions on lifestyle factors could possibly improve motivation for weight loss.

The HMLT automatically divides original data to increase the mining accuracy of patterns, trends, and rules in the data. Thus, a machine learning model can provide optimal predictive equations automatically. In this study, we constructed a system that automatically identified targets whose weight changed greatly after lifestyles factors had changed. Previous reviews showed that many factors influence body weight (38, 39), suggesting that the effectiveness of standardized treatment used in clinical settings among individuals. Our findings indicated that individualized guidance may help achieve weight loss efficiently in clinical practice.

Thus far, the most important criticism of artificial intelligence is its clinical context as a “black box” which cannot easily explain the reasons for and background of results learned by the model, which could be a fatal barrier for its current use in clinical practice and care, which is required to be based on evidence based medicine (42–44). Indeed, there are some studies that have used artificial intelligence to predict weight at a single point, but those models cannot be explained in a way that humans can understand or predict predicted body weight only once (26, 27). The HMLT is epoch-making in that it can be understood by providers and recipients of medical services. In fact, this method can construct predictive models from data quickly and automatically. In addition, large amounts of time are not required to create and verify complicated predictive formulas. Thus, the workload of medical staff would not only be reduced but benefits would accrue to patients.

Our study had several limitations. First, we could not obtain certain information, such as information on family members and socioeconomic status, that would affect body weight (3, 4). Second, we included only participants who underwent a medical examination. Third, the information on diet and exercise was assessed using a self-reported questionnaire. Fourth, the fact that the study population was exclusively ethnic Japanese could limit wider applicability of the results.

In conclusion, the machine learning model could predict weight change over a period of 3 years. Our model automatically identified groups whose lifestyles have a profound impact on weight loss and the factors that influence body weight in individuals. Those findings suggested the possibility that a machine learning model could support individualized guidance for weight management in clinical settings.

## Data availability statement

The datasets presented in this article are not readily available due to proprietary reasons. Queries regarding the datasets should be directed to kafujihara-dm@umin.ac.jp.

## Ethics statement

The studies involving human participants were reviewed and approved by Niigata University (2020-0003). The patients/participants provided their written informed consent to participate in this study.

## Author contributions

HS: full access to all data in the study and takes responsibility for the integrity of the data and the accuracy of the data analysis. KF, MY, CH, MI, and TY: study concept and design. KF and KK: acquisition of data. KF, HT, HN, YS, and KT: analysis and interpretation of data, drafting of the manuscript. KF, HN, SK, KK, and HS: critical revision of the manuscript for important intellectual content. SK, KK, and HS: study supervision. All authors contributed to the article and approved the submitted version.

## Funding

This work was supported in part by the Japan Society for the Promotion of Science and the Ministry of Health, Labor and Welfare, Japan (19H04028, 21 K11569). The sponsor or funding organization had no role in the design or conduct of this research.

## Conflict of interest

HT, HN, YS, and KT were employed by NEC Solution Innovators, Ltd.

The remaining authors declare that the research was conducted in the absence of any commercial or financial relationships that could be construed as a potential conflict of interest.

## Publisher’s note

All claims expressed in this article are solely those of the authors and do not necessarily represent those of their affiliated organizations, or those of the publisher, the editors and the reviewers. Any product that may be evaluated in this article, or claim that may be made by its manufacturer, is not guaranteed or endorsed by the publisher.

## Supplementary material

Supplementary Figure 1 Schematic diagram of our heterogeneous mixture learning technology. Heterogeneous mixture learning technology is a method to automatically divide original data to increase the mining accuracy of patterns, trends, and rules in the data. For example, for the hypotheses that the data contain a mixture of nonlinear and linear relationships **(A)** and heterogeneous mixture learning technology can divide the data into two groups **(B)**. However, for the hypothesis of a mixture of multiple linear relationships **(C)**, heterogeneous mixture learning technology automatically generates three groups.

Supplementary Figure 2 Trend of future body weight according to **(A,C)** breakfast intake (yes or no) and **(B,D)** walking speed (fast or not). **(A,B)** show people with a high body mass index at baseline (formula 1 or 2). **(C,D)** show younger people with a low body mass index at baseline (formula 5). The first two show measured values and the latter three show predicted values for body weight.

## Abbreviations

BMI: body mass index
HMLT: heterogeneous mixture learning technology
RMSE: root mean square error
