# Supplementary figures and images for: Machine learning approach to predict body weight in adults
Source: Front Public Health. 2023 Jun 15;11:1090146. doi: 10.3389/fpubh.2023.1090146 (PMC10308016; doi:10.3389/fpubh.2023.1090146)

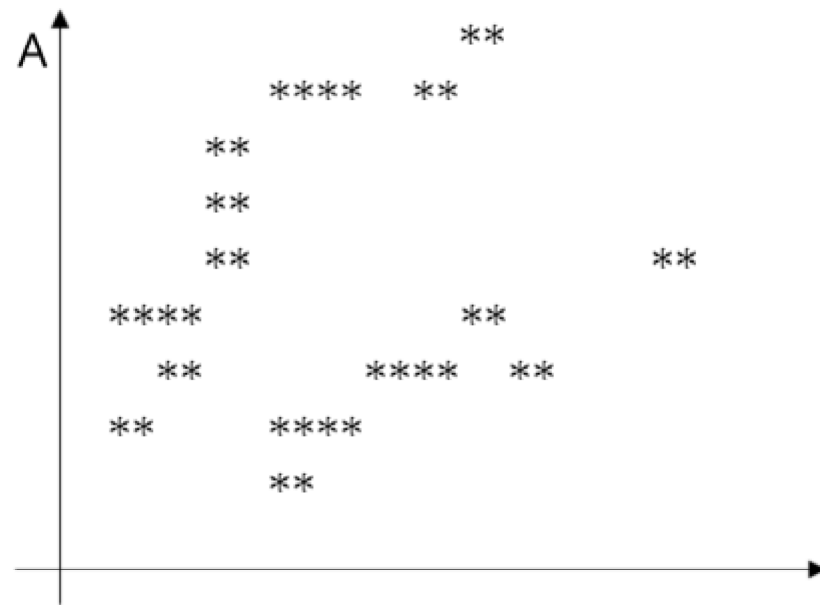

Supplement: Supplementary Figure 1 — Schematic diagram of our heterogeneous mixture learning technology. Heterogeneous mixture learning technology is a method to automatically divide original data to increase the mining accuracy of patterns, trends, and rules in the data. For example, for the hypotheses that the data contain a mixture of nonlinear and linear relationships (A) and heterogeneous mixture learning technology can divide the data into two groups (B). However, for the hypothesis of a mixture of multiple linear relationships (C), heterogeneous mixture learning technology automatically generates three groups. [file Image_1.pdf]

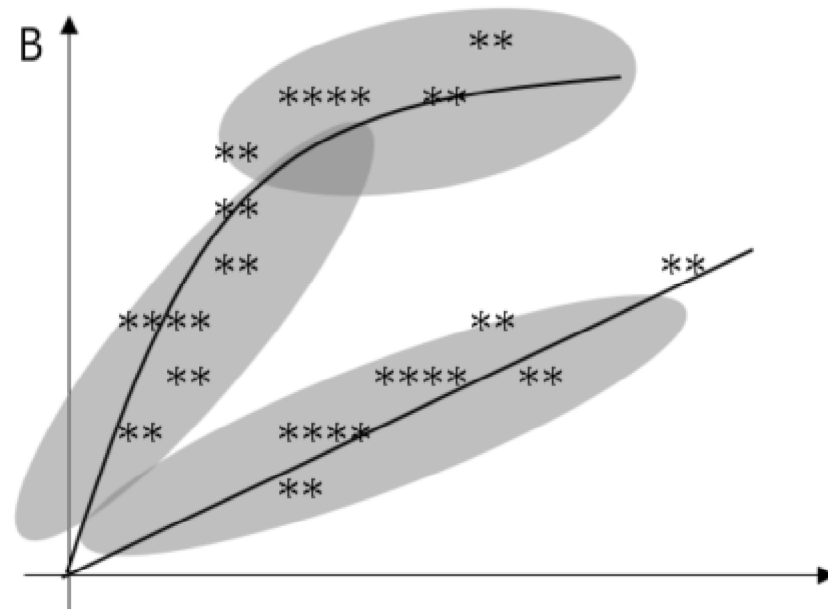

Supplement: Supplementary Figure 2 — Trend of future body weight according to (A,C) breakfast intake (yes or no) and (B,D) walking speed (fast or not). (A,B) show people with a high body mass index at baseline (formula 1 or 2). (C,D) show younger people with a low body mass index at baseline (formula 5). The first two show measured values and the latter three show predicted values for body weight. [file Image_2.pdf]

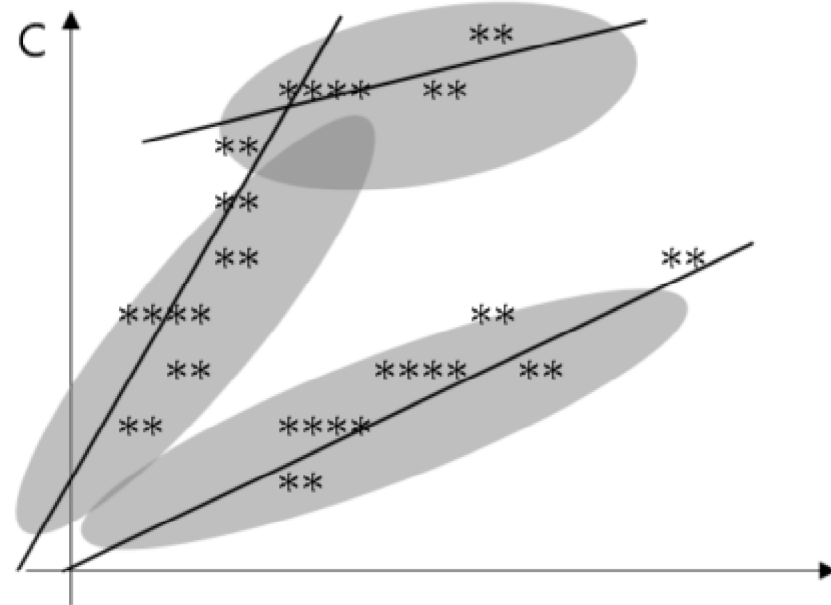

Supplement: Supplementary file 3 [file Image_3.pdf]

A

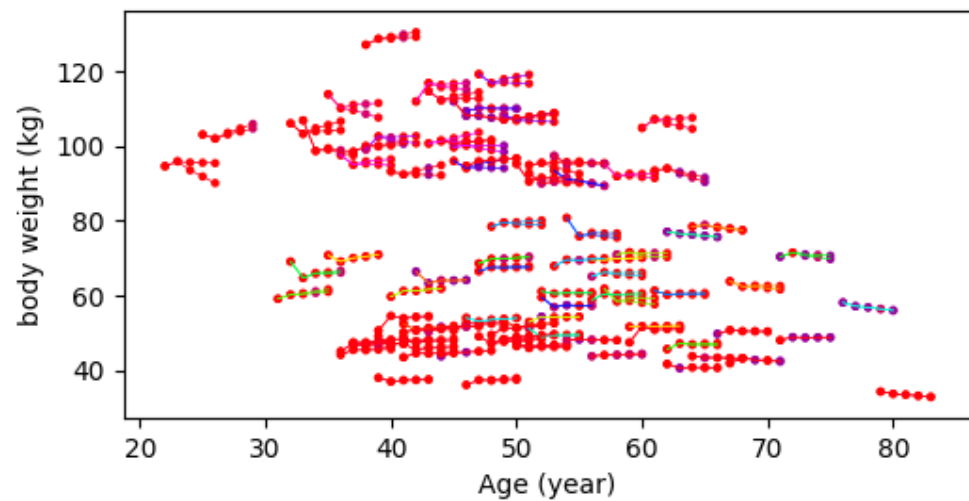

Supplement: Supplementary file 4 [file Image_4.pdf]

B

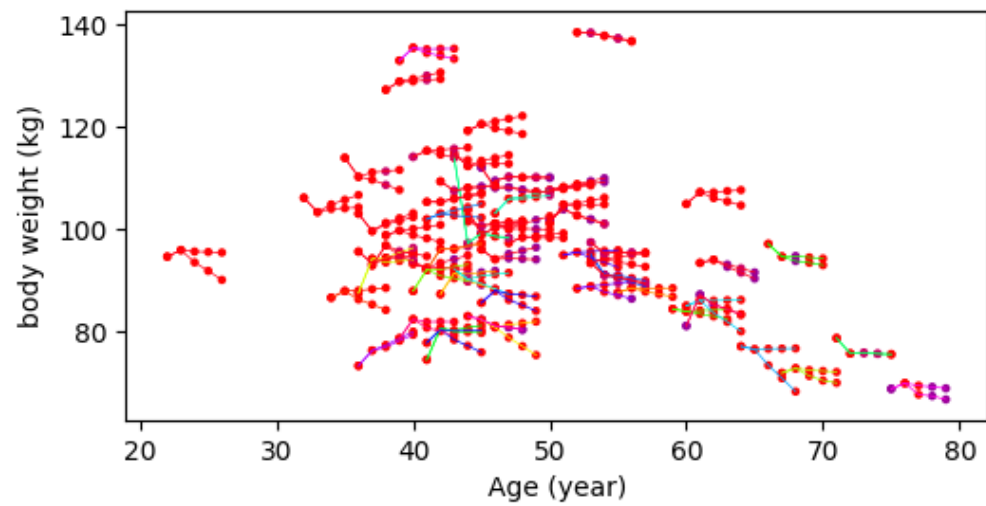

Supplement: Supplementary file 5 [file Image_5.pdf]

C

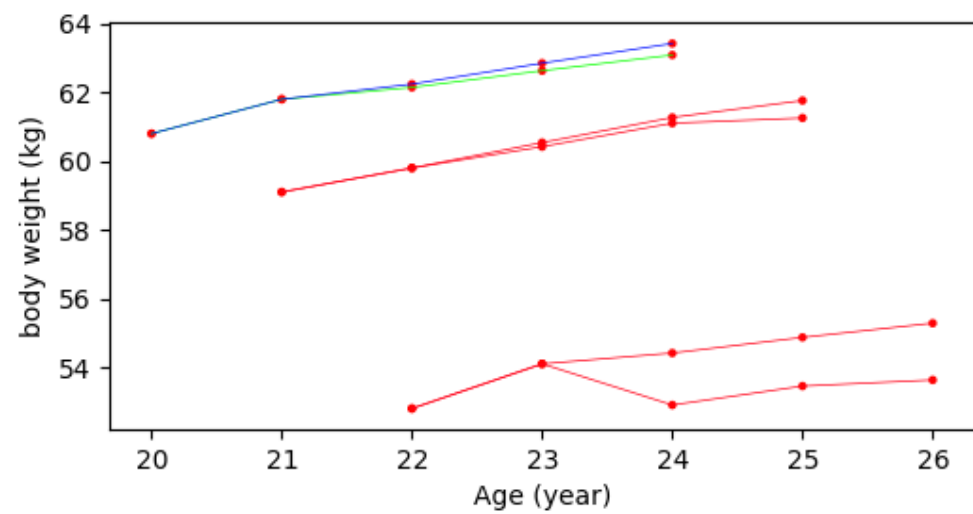

Supplement: Supplementary file 6 [file Image_6.pdf]

D

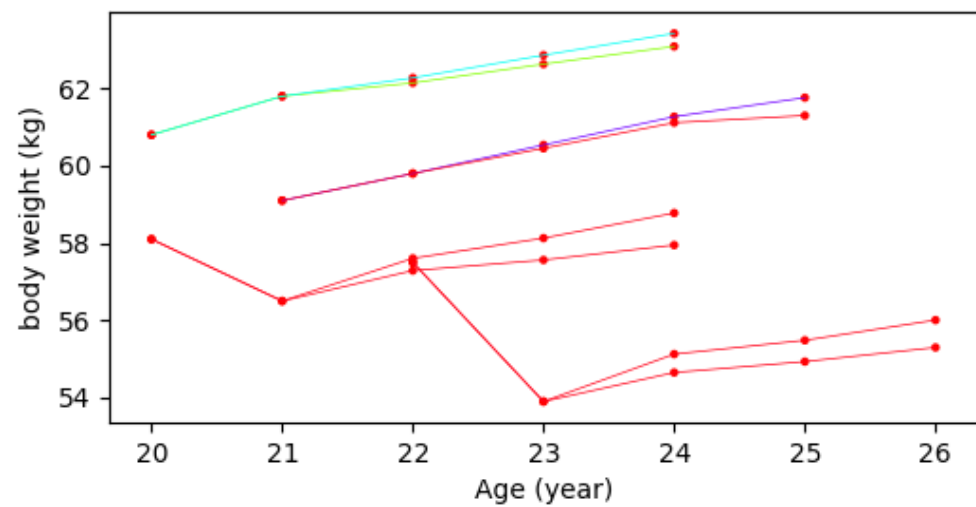

Supplement: Supplementary file 7 [file Image_7.pdf]
