# Supplementary material for: Machine learning approach to predict body weight in adults
Source: Front Public Health. 2023 Jun 15;11:1090146. doi: 10.3389/fpubh.2023.1090146 (PMC10308016; doi:10.3389/fpubh.2023.1090146)
Supplement: Supplementary file 8 [file Table_1.docx]

**Supplemental Table 1.** Multiple regression analysis in prediction of body weight

| Explanatory variables | Intention to improve lifestyle * | | | standardized beta coefficient |
| --- | --- | --- | --- | --- |
|  | 1 | 2 | 3 |  |
| Weight at baseline |  |  |  | 0.9972 |
| Difference in weight between one years ago and baseline |  |  |  | -0.1967 |
| Height at baseline |  |  |  | 0.0047 |
| Sex (female) |  |  |  | -0.0249 |
| Age at baseline |  |  |  | -0.0127 |
| Interview at baseline. Do you want to use the opportunity of health instructions for improvement of your life habits? (No) |  |  |  | 0.0451 |
| Interview at predictive year. Do you want to use the opportunity of health instructions for improvement of your life habits? (No) |  |  |  | -0.0162 |
| Interview at baseline. Are you a heavy smoker ? (No) |  |  |  | -0.6218 |
| Interview at predictive year. Are you a heavy smoker ? (Yes) | 〇 |  |  | -0.0489 |
|  |  | 〇 |  | -0.3202 |
|  |  |  | 〇 | 0.6617 |
| Interview at predictive year. Are you a heavy smoker ? (No) |  | 〇 |  | 0.6210 |
|  |  |  | 〇 | 0.2655 |
| Interview at baseline. Drinking frequency and quantity |  |  |  | -0.0037 |
| Interview at predictive year. Drinking frequency and quantity |  |  |  | 0.0027 |
| Interview at baseline. Is your walking speed faster than the speed of those of your age and sex? (No) |  |  |  | -0.0405 |
| Interview at predictive year. Is your walking speed faster than the speed of those of your age and sex? (No) | 〇 |  |  | 0.0409 |
| Interview at predictive year. Is your walking speed faster than the speed of those of your age and sex? (Yes) |  | 〇 |  | 0.0244 |
|  |  |  | 〇 | 0.1244 |
| Interview at baseline. Do you walk or have any equivalent amount of physical activity more than one hour a day in your daily life? (No) |  |  |  | -0.0962 |
| Interview at predictive year. Do you walk or have any equivalent amount of physical activity more than one hour a day in your daily life? (No) | 〇 |  |  | 0.1353 |
| Interview at predictive year. Do you walk or have any equivalent amount of physical activity more than one hour a day in your daily life? (Yes) |  | 〇 |  | -0.0343 |
|  |  |  | 〇 | -0.0999 |
| Interview at baseline. Do you perform exercise of moderate intensity at least twice a week for 30 min over a period of one year? (No) |  |  |  | -0.1486 |
| Interview at predictive year. Do you perform exercise of moderate intensity at least twice a week for 30 min over a period of one year? (No) | 〇 |  |  | 0.1318 |
| Interview at predictive year. Do you perform exercise of moderate intensity at least twice a week for 30 min over a period of one year? (Yes) |  | 〇 |  | -0.0253 |
|  |  |  | 〇 | 0.0992 |
| Interview at baseline. Do you skip breakfast more than 3 times a week? (No) |  |  |  | -0.1600 |
| Interview at predictive year. Do you skip breakfast more than 3 times a week? (No) | 〇 |  |  | 0.0580 |
| Interview at predictive year. Do you skip breakfast more than 3 times a week? (Yes) |  | 〇 |  | -0.0753 |
|  |  |  | 〇 | -0.1957 |
| Interview at baseline. Is your eating speed quicker than others? (normal) |  |  |  | 0.0496 |
| Interview at baseline. Is your eating speed quicker than others? (late) |  |  |  | -0.0074 |
| Interview at predictive year. Is your eating speed quicker than others? (quicker) |  | 〇 |  | -0.0240 |
|  |  |  | 〇 | -0.1425 |
| Interview at predictive year. Is your eating speed quicker than others? (normal) | 〇 |  |  | -0.0919 |
|  |  | 〇 |  | -0.1549 |
|  |  |  | 〇 | -0.1270 |
| Interview at predictive year. Is your eating speed quicker than others? (late) | 〇 |  |  | -0.1511 |
| Interview at baseline. Do you eat supper two hours before bedtime more than 3 times a week? (No) |  |  |  | 0.0237 |
| Interview at predictive year. Do you eat supper two hours before bedtime more than 3 times a week? (No) | 〇 |  |  | -0.0891 |
| Interview at predictive year. Do you eat supper two hours before bedtime more than 3 times a week? (Yes) |  | 〇 |  | 0.0486 |
|  |  |  | 〇 | 0.1669 |
| I Interview at baseline. Do you eat snacks or drink sweet beverage between meals? (No) |  |  |  | 0.0976 |
| Interview at predictive year. Do you eat snacks or drink sweet beverage between meals? (No) | 〇 |  |  | -0.1060 |
| Interview at predictive year. Do you eat snacks or drink sweet beverage between meals? (Yes) |  | 〇 |  | 0.1121 |
|  |  |  | 〇 | 0.1650 |
| Interview at baseline. Do you sleep well and enough? (No) |  |  |  | 0.0906 |
| Interview at predictive year. Do you sleep well and enough? (No) | 〇 |  |  | -0.0593 |
| Interview at predictive year. Do you sleep well and enough? (Yes) |  | 〇 |  | -0.0728 |
|  |  |  | 〇 | 0.0900 |
| bias |  |  |  | 0.4271 |

*Do you want to improve your life habits such as eating and exercise? 1: No, 2: already trying to improve (less than 6 months), 3: already trying to improve (over 6 months)
